# The association of parameters of body composition and laboratory markers with the severity of hypertriglyceridemia-induced pancreatitis

**DOI:** 10.1186/s12944-021-01443-7

**Published:** 2021-02-11

**Authors:** Lifang Chen, Yingbao Huang, Huajun Yu, Kehua Pan, Zhao Zhang, Yi Man, Dingyuan Hu

**Affiliations:** 1 Department of Radiology, the First Affiliated Hospital of Wenzhou Medical University, Wenzhou, China; 2 The Center of Diagnosis and Treatment of Pancreatitis, the First Affiliated Hospital of Wenzhou Medical University, Wenzhou, China; 3 Department of Gastroenterology, the Second Affiliated Hospital of Wenzhou Medical University, Xue Yuan Xi Lu 109, Lucheng District, Wenzhou, 325027 China

**Keywords:** Hypertriglyceridemia-induced pancreatitis, Computed tomography, Body composition, Albumin, Severity, Apolipoprotein A-I, Pancreatic necrosis

## Abstract

**Background:**

Hypertriglyceridemia has arisen as the third leading cause of acute pancreatitis. This study aimed at exploring the association between the severity of hypertriglyceridemia-induced pancreatitis (HTGP) and computed tomography (CT)-based body composition parameters and laboratory markers.

**Methods:**

Laboratory and clinical parameters were collected from 242 patients with HTGP between 2017 and 2020. Severity of HTGP was evaluated by original or modified CT severity index. Body composition parameters such as area and radiodensity of muscle, subcutaneous adipose tissue and visceral adipose tissue were calculated by CT at the level of third lumbar vertebra. Parameters were compared between mild and moderately severe to severe HTGP. Uni-variate and multi-variate Logistic regression analyses were employed to assess the risk factors of the severity of HTGP.

**Results:**

Seventy patients (28.9%) presented with mild HTGP. Body mass index, waist circumference and all CT-based body composition parameters differed between male and female patients. None was associated with the severity of HTGP, neither in males nor in females. Receiver operating characteristic curves showed that areas under the curves of apolipoprotein A-I and albumin to predict the severity of HTGP were 0.786 and 0.759, respectively (all *P* < 0.001). Uni-variate and further multi-variate Logistic regression analysis confirmed that low serum albumin (< 35 g/L, *P* = 0.004, OR = 3.362, 95%CI = 1.492–8.823) and apolipoprotein A-I (< 1.1 g/L, *P* < 0.001, OR = 5.126, 95%CI = 2.348–11.195), as well as high C-reactive protein (> 90 mg/L, *P* = 0.005, OR = 3.061, 95%CI = 1.407–6.659) and lipase (*P* = 0.033, OR = 2.283, 95%CI = 1.070–4.873) were risk factors of moderately severe to severe HTGP. Levels of albumin, apolipoprotein A-I, C-reactive protein and lipase were also associated with the length of hospital stay (all *P* < 0.05). Besides, low serum albumin, low-density lipoprotein cholesterol and high radiodensity of subcutaneous adipose tissue were significant risk factors of pancreatic necrosis in patients with HTGP (all *P* < 0.05).

**Conclusions:**

Low serum albumin and apolipoprotein A-I, and high C-reactive protein and lipase upon admission were associated with a more severe type of HTGP and longer hospital stay for these patients. Albumin and apolipoprotein A-I may serve as novel biomarkers for the severity of HTGP. However, none of the body composition parameters was associated with the severity of HTGP.

## Background

Acute pancreatitis (AP) is the inflammation of the pancreas with unpredictable clinical outcome. Although most acute pancreatitis cases are mild and can be cured successfully, some can lead to local complications, systematic inflammatory response syndrome and organ failure, which are associated with a substantial mortality. Gallstones and alcohol abuse are currently the most frequent causes of AP. Notably, the proportion of hypertriglyceridemia (HTG)-induced pancreatitis (HTGP) in AP is increasing during the decades [1], and now ranked as the third leading cause for AP [2, 3]. HTGP accounts for approximately 9% of all cases and as much as 56% of AP cases during pregnancy [4]. It is associated with a similar clinical course as other forms of AP, only distinguished by initially being HTG [5]. Although debated, HTGP tends to be more severe than AP caused by other etiological factors [4]. It has been postulated that HTGP may specifically benefit from plasmapheresis [4], which is the most direct and life-saving therapy in severe episodes.

HTG is defined as fasting plasma triglycerides (TGs) over 1.7 mmol/L or non-fasting TGs over 2.0 mmol/L [6]. It is classified as moderate HTG with TGs level ranging from 1.7–5.6 mmol/L, or severe HTG when TGs are ≥5.6 mmol/L. In patients with moderate HTG, identification and corresponding management of secondary factors such as Diabetes Mellitus, hypothyroidism and chronic liver disease is highly recommended [7]. Statin therapy should be initiated in those with persistent HTG in order to reduce atherosclerotic cardiovascular risk when secondary factors have been addressed. In patients with severe HTG, chylomicrons begin to be involved in the carriage of TGs, while their increase in plasma confers a higher risk of acute pancreatitis [8]. This risk increases with the degree of elevation of TGs and pancreatitis typically occurs when TGs are over 11.3 mmol/L. Therefore, prevention of pancreatitis is a key target for the management of severe HTG. To reduce the risk of HTGP, further treatments for severe HTG include a very-low-fat diet, restricted carbohydrate and alcohol intake, fibrates and Omega-3 fatty acids [8, 9]. Most patients with severe HTGs have a genetic component. For instance, familial chylomicronemia syndrome (FCS) is a rare monogenic disorder responsible for extremely high levels of plasma TGs, putting patients at a very high risk for acute pancreatitis [10].

The precise pathophysiology underlying HTGP remain unclear. However, it is generally believed that the deposition of free fatty acids hydrolyzed by pancreatic lipase from triglyceride (TG) drives the occurrence of the disease [11]. The fatty acids can be bound to serum albumin, whereas the exceeded free fatty acids exert a detergent-like role and attacks platelets, vascular endothelium, and acinar cells. Although some studies have recognized the effect of albumin levels on severity and organ failure in AP, it has not been included as a risk factor of AP in most studies and in clinical practice. To our knowledge, there are no studies investigating the association of serum albumin with HTGP, despite that serum albumin might be more closely linked to the deposition of free fatty acids and the severity of HTGP.

Since the prognosis and clinical intervention of AP differs largely in mild AP and severe AP, the early prediction of the severity in AP has been a great concern and research focus for clinicians. Predictive parameters, including clinical, laboratory and radiological markers, as well as various scoring systems, have been proposed. Current guidelines recommend that persistent systemic inflammatory response syndrome or organ failure for at least 48 h is predictive of the severity of AP [12]. Other recognized risk factors for development of severe AP include elevated hematocrit, blood urea nitrogen and creatinine and body mass index [13, 14]. Several scoring systems, such as Acute Physiology and Chronic Health Evaluation II (APACHE II), the Bedside Index for Severity in Acute Pancreatitis (BISAP), and Ranson’s Criteria have proven their correlation with the severity of AP, but their clinical utilities are still limited due to the low predictive values [15, 16]. Computed tomography (CT) on admission has a similar predictive accuracy for the severity of AP as clinical scoring systems [17]. Although not recommended by the guidelines [12], abdominal CT upon admission is often conducted in AP in real-world practice, especially in the emergent situation to exclude differential diagnoses. Contrast-enhanced CT is advantageous to delineate pancreatic or peripancreatic fluid collections and necrosis. It is the most applied method in the clinic to re-evaluate the severity of cases in potential deterioration.

Recent studies have suggested that the severity of AP is also linked to some radiological parameters describing body composition [18]. Larger peripancreatic volume of visceral adipose tissue (VAT) was found associated with severe AP [19] and high visceral fat with low skeletal muscle volume strongly correlated with AP severity [20]. A recent systematic review, which included 11 studies, concluded that VAT is an important prognostic indicator for the severity of AP and may be incorporated into the prognostic scoring systems of AP [21]. Nevertheless, muscle radiodensity, rather than VAT and subcutaneous adipose tissue (SAT), correlated with severe acute pancreatitis [22]. Notably, by stratifying AP into HTGP and non-HTGP, it was revealed that VAT and VAT/SAT were valuable factors for predicting the severity in HTGP, but not in non-HTGP [23].

With a focus on HTGP, a subgroup of AP with rising incidence, this study aimed at exploring body composition parameters and clinical risk factors for the severity of HTGP, which may help predict the prognosis of HTGP.

## Methods

### Patients

A total of 242 hospitalized patients with HTGP from the First Affiliated Hospital of Wenzhou Medical University between July 2017 and August 2020 were retrospectively studied. The diagnosis of AP was established according to the Revised Atlanta Definitions of AP [24], when two of the following three characteristics were met: (1) the symptoms of abdominal pain were consistent with AP, (2) the levels of amylase and/or lipase were at least 3 times above the upper limit of the normal, and (3) abdominal imaging was consistent with changes in AP. HTGP was considered in AP patients when the level of serum TGs was (1) over 11.3 mmol/L; (2) between 5.65 mmol/L and 11.3 mmol/L, milky serum, with no other etiology of AP; (3) not tested, but the patient, without any known etiology, had previously been diagnosed as HTGP. The inclusion criteria of patients in this study were (1) the diagnosis of HTGP was established; (2) abdominal CT scanning 72 h before the occurrence of symptom; exclusion criteria were (1) poor CT imaging of the abdomen; (2) indication of biliary, alcoholic, autoimmune, drug-induced or pancreatic tumor-related etiology of AP; (3) pregnancy. The study protocol was approved by the ethics committee of the First Affiliated Hospital of Wenzhou Medical University (KY2019–011). Participant informed consent was waived given the retrospective study design.

Contrast-enhanced CT scannings of abdomen were performed in 177 (73.1%) of the studied patients when deterioration of the disease occurred during the course of the disease. The severity of HTGP was evaluated by modified CT severity index (MCTSI) if contrast-enhanced CT scanning had been conducted, otherwise by the CT severity index based on unenhanced CT scanning.

Clinical parameters of each patient were retrieved from the Electronic Health Record System, including sex, gender, body mass index, length of hospital stay, and C-reactive protein (CRP), serum albumin, amylase, total cholesterol, low-density lipoprotein cholesterol (LDL-C), high-density lipoprotein cholesterol (HDL-C), apolipoprotein A-I (apoA-I), apolipoprotein B (apoB) and lipoprotein upon the admission (Table 1). CRP was determined by turbidimetric inhibition immunoassay, while all other plasma parameters were determined by Siemens ADVIA 2400 automatic biochemical analyzer. The levels of CRP, albumin and TGs were categorized as high and low with the cutoff of 90 mg/L, 35 g/L and 22.4 mmol/L [25], respectively. APACHE II, BISAP, Ranson and Marshall scorings of the patients were calculated when possible.

**Table 1:** Comparisons of laboratory parameters and clinical data in patients with mild and moderately severe to severe hypertriglyceridemia-induced pancreatitis

Variables	HTGP	Mild HTGP	Moderately severe to Severe HTGP	*P* values	Missing values
Sex				0.141	
Male	193	60 (85.7)	133 (77.3)		
Female	49	10 (14.3)	39 (22.7)		
Age (years)	40 (34–47)	42 (35–49)	39 (33–46)	0.101	
CRP (mg/L)				**< 0.001**	21
< 90	91	43 (69.4)	48 (30.2)		
≥ 90	130	19 (30.6)	111 (69.8)		
Albumin (g/L)	36.54 ± 6.56	40.38 ± 4.89	34.98 ± 6.52	**< 0.001**	
< 35	103	11 (15.7)	92 (53.5)	**< 0.001**	
≥ 35	139	59 (84.3)	80 (46.5)		
Triglyceride (mmol/L)	12.74 (8.18–25.12)	10.64 (6.69–21.50)	13.34 (9.45–26.10)	**0.008**	12
High (< 22.4 mmol/L)	163	50 (76.9)	113 (68.5)	0.205	
Very high (≥22.4 mmol/L)	67	15 (23.1)	52 (31.5)		
Amylase (U/L)	121 (67–269)	90 (51–216)	133 (73–314)	**0.027**	
Lipase (U/L)	234 (112–612)	151 (84–404)	263 (126–664)	**0.003**	13
Total cholesterol (mmol/L)	8.42 (6.31–12.19)	7.24 (5.95–10.27)	9.11 (6.53–13.14)	**0.006**	
HDL-C (mmol/L)	0.60 (0.46–0.79)	0.76 (0.62–0.87)	0.56 (0.43–0.70)	**< 0.001**	1
LDL-C (mmol/L)	2.06 (1.59–1.92)	1.96 (1.57–2.46)	2.13 (1.59–2.97)	0.231	1
Non-HDL-C (mmol/L)	8.04 (5.47–11.53)	6.48 (5.13–9.52)	8.50 (5.80–12.56)	**0.004**	1
ApoA-I (g/L)	0.84 (0.67–1.09)	1.10 (0.84–1.30)	0.78 (0.66–0.97)	**< 0.001**	7
ApoB (g/L)	0.81 (0.49–1.29)	0.80 (0.48–1.17)	0.81 (0.48–1.35)	0.294	36
Lipoprotein (mg/L)	48.5 (26.8–115.3)	48.0 (30.0–86.0)	49.0 (23.0–129.0)	0.830	8
Length of hospital stay (days)	13 (9–20)	9 (7–11.5)	17 (11–21)	**< 0.001**	
< 14	131	61 (88.4)	70 (40.7)	**< 0.001**	
≥ 14	110	8 (11.6)	102 (59.3)		

*ApoA-I* apolipoprotein A-I, *ApoB* apolipoprotein B, *CRP* C-reactive protein, *HDL-C* high-density lipoprotein cholesterol, *HTGP* hypertriglyceridemia-induced pancreatitis, *LDL-C* low-density lipoprotein cholesterol

### CT scanning and body composition parameters

CT scanning was performed by 64-slice spiral CT scanner (Lightspeed VCT, GE healthcare, USA) or Aquilion ONE 320 Slice CT scanner (Toshiba, Japan). The scanning covered the whole abdomen. The slice thickness was 0.625 mm for 64-slice spiral CT scanner (pitch 0.984, single-turn spiral time 0.5 s, 100 kV, 500 mA) and 0.5 mm for the 320 Slice CT scanner (single-turn spiral time 0.5 s, 100 kV, 300 mA). For contrast-enhanced CT, an auto-injector was used to inject 60 mL of non-ionic contrast agent (iopromide 300mgI/mL) and 30 mL of saline at a speed of 4.0 mL/s, and the scanning was triggered intelligently by monitoring of the abdominal aorta, the arterial phase was delayed for 30–35 s, and the portal phase was delayed for 60–70 s. The imaging data were transferred to the post-processing workstation (Version 4.5, GE healthcare).

Body composition parameters were measured based on the non-enhanced CT scanning. Muscle and adipose tissue at the level of third lumbar vertebra (L3) with supine position were analyzed by Image J software [26]. A range of − 29 to 150 Hounsfield units (HU) was set to highlight muscle, a range of − 190 to − 30 HU was set to highlight SAT, and − 150 to 50 HU for VAT. The area and density of the region of interest (ROI) were calculated automatically. Waist circumference was measured at the umbilical plane. CT measurements were performed by 2 experienced radiologists blinded to the clinical information. Re-measurement took place when disagreement of the measurements occurred.

### Statistical analysis

Parameters between mild and moderately severe to severe HTGP were compared. Comparisons of categorical data were performed using Chi-square test or Fisher’s exact test. Continuous data were expressed as mean ± standard deviation and compared by the student’s t test when normal distribution was justified, otherwise expressed as median (interquartile range) and compared by Mann Whitney U test. Uni-variate and multi-variate Logistic regression analyses were employed to assess the risk factors for the severity of HTGP. Variables with *P*-values less than 0.1 in univariate analysis were included for the multi-variate analysis with a backward method. To test the predictive capacity of the measured parameters, receiver operating characteristic (ROC) curves with corresponding areas under the curves (AUC) were calculated. A *P*-value of < 0.05 was considered statistically significant. All the statistics were performed using SPSS 18.0 (IBM. SPSS Statistics for Windows, USA).

## Results

### Association of laboratory parameters and clinical data with the severity of HTGP

To investigate the association of the severity of HTGP with laboratory parameters and clinical data, comparisons were made between patients with mild HTGP and those with moderately severe to severe HTGP. Age and gender distribution showed no difference between the two groups (all *P* > 0.05). In comparison, the levels of serum CRP, amylase and lipase, TGs, total cholesterol and non-HDL-C were significantly higher in patients with moderately severe to severe HTGP than those with mild HTGP (all *P* < 0.05). However, concentrations of serum albumin (mean 34.98 vs. 40.38 g/L), HDL-C (median 0.56 vs. 0.76 mmol/L), apoA-I (median 0.78 vs. 1.10 g/L) were significantly lower in the severe group (all *P* < 0.001, Table 1).

**Fig. 1:**
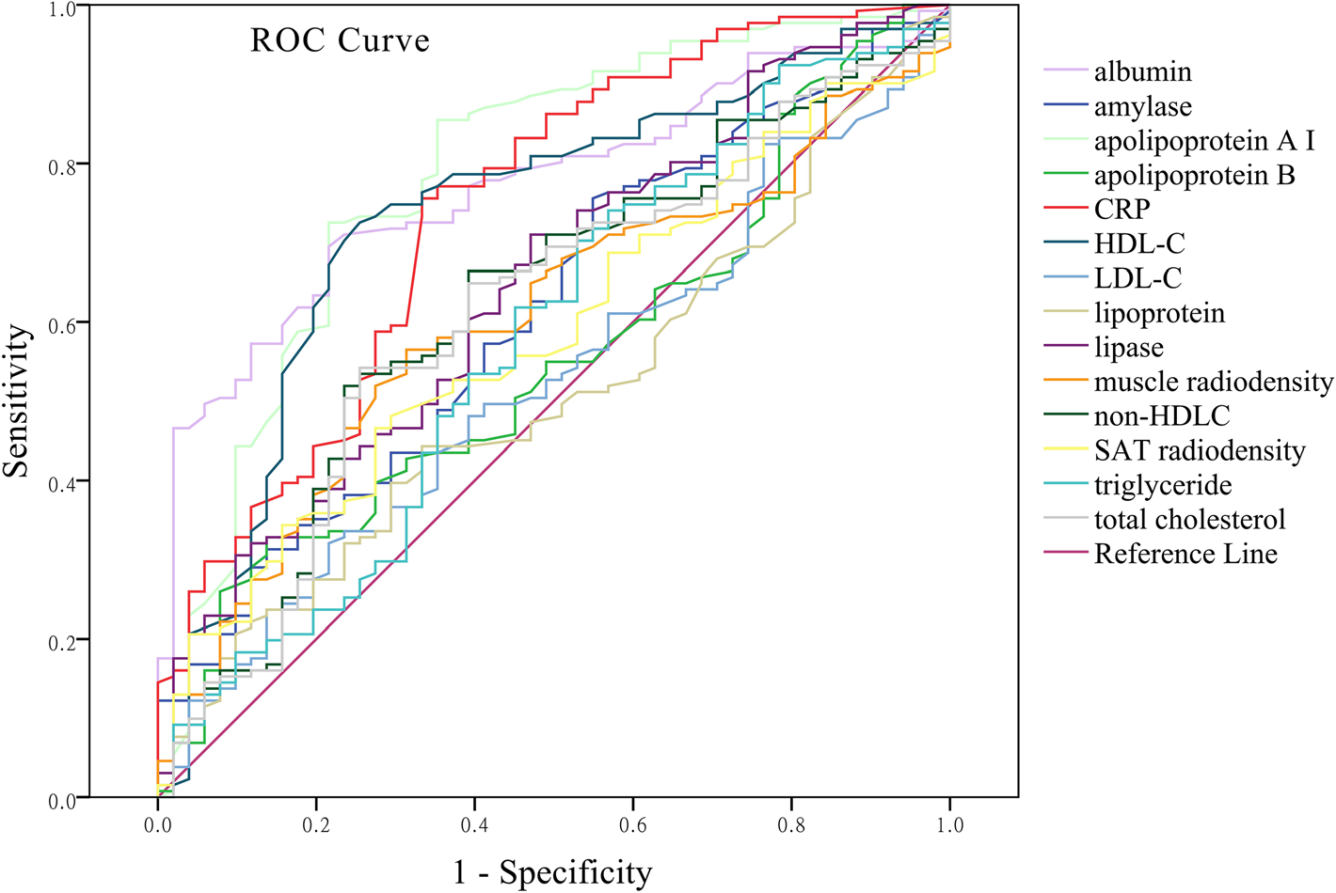
Receiver operating characteristic curves of parameters for the predictive capacity for severity of HTGP. Apolipoprotein A-I (*P* < 0.001, AUC = 0.786), albumin (*P* < 0.001, AUC = 0.759), C-reactive protein (CRP) (*P* < 0.001, AUC = 0.743) and high-density lipoprotein cholesterol (HDL-C) (*P* < 0.001, AUC = 0.735) showed the most statistical significance for predicting the severity of HTGP. AUC: areas under the curves; HTGP: hypertriglyceridemia-induced pancreatitis; LDL-C: low-density lipoprotein cholesterol; SAT: subcutaneous adipose tissue

ROC analysis depicted the AUCs of parameters for the prediction of HTGP severity (Fig. 1). ApoA-I (*P* < 0.001, AUC = 0.786), albumin (*P* < 0.001, AUC = 0.759), CRP (*P* < 0.001, AUC = 0.743) and HDL-C (*P* < 0.001, AUC = 0.735) showed the most statistical significance for the prediction of HTGP severity. With a balanced cutoff of 1.1 g/L, apoA-I predicted the severity of HTGP with a sensitivity and specificity of 0.725 and 0.784, respectively. A cutoff of 36.65 g/L for albumin concentration achieved the sensitivity of 0.786 and specificity of 0.651. With the cutoff of 35.0 g/L, the sensitivity and specificity were 0.843 and 0.535, respectively. AUCs for other parameters were: muscle radiodensity (0.625, *P* = 0.004), SAT radiodensity (0.580, *P* = 0.066), triglyceride (*P* = 0.027, AUC = 0.596), amylase (0.613, *P* = 0.018), lipase (0.645, *P* = 0.002), total cholesterol (0.611, *P* = 0.020), LDL-C (0.524, *P* = 0.617), non-HDL-C (0.621, *P* = 0.011), apoB (0.553, *P* = 0.264), lipoprotein (0.512, *P* = 0.797).

### Body composition parameters were divergent between male and female patients with pancreatitis

Notably, apparent differences existed between male and female patients regarding all the body composition parameters studied in this study, including BMI, muscle area, muscle radiodensity, SAT area, SAT radiodensity, VAT area, VAT area/total adipose tissue area and waist circumference (all *P* < 0.05). Therefore, stratified comparisons in either male or female patients were performed. The mean values of each parameter in male and female patients with HTGP are shown in Table 2. Consequently, the muscle radiodensity (mean 42.9 vs. 44.8 HU, *P* = 0.020) was lower while SAT radiodensity (mean − 95.0 vs. -97.7 HU, *P* = 0.009) was higher in male patients with moderately severe or severe HTGP than in those with mild HTGP. For the subsequent analyses, all body composition parameters were categorized as high and low subgroups using corresponding median value in males or females as the cutoff. Uni-variate Logistic regression analysis indicated that none of the body composition parameters was associated with the severity of hypertriglyceridemia-induced pancreatitis, neither in male nor in female patients (all *P* > 0.05, Table 3).

**Table 2:** Comparisons of body composition parameters in patients with mild and moderately severe to severe hypertriglyceridemia-induced pancreatitis

Variables	HTGP	Mild HTGP	Moderately severe to Severe HTGP	*P* values
Body mass index				
Male	26.3 ± 3.3	26.3 ± 2.8	26.3 ± 3.5	0.885
Female	24.5 ± 3.8	24.4 ± 5.8	24.5 ± 3.2	0.912
Muscle area (mm^2^)				
Male	166.3 ± 27.5	165.4 ± 27.6	166.7 ± 27.5	0.763
Female	110.1 ± 16.6	107.7 ± 13.0	110.7 ± 17.5	0.612
Muscle radiodensity (HU)				
Male	43.5 ± 6.0	44.8 ± 4.8	42.9 ± 6.4	**0.020**
Female	39.6 ± 5.8	42.0 ± 4.6	39.0 ± 6.0	0.158
SAT area (mm^2^)				
Male	123.5 ± 45.0	1213 ± 45.3	124.5 ± 49.3	0.664
Female	167.5 ± 64.5	157.6 ± 80.0	170.0 ± 61.0	0.592
SAT radiodensity (HU)				
Male	−95.8 ± 7.7	−97.7 ± 5.7	−95.0 ± 8.4	**0.009**
Female	−99.8 ± 5.4	−101.2 ± 3.9	−99.5 ± 5.7	0.368
VAT area (mm^2^)				
Male	181.9 ± 66.9	187.9 ± 63.7	179.2 ± 68.4	0.405
Female	122.8 ± 52.6	105.5 ± 41.8	127.2 ± 54.6	0.248
VAT area /TAT area				
Male	0.589 ± 0.110	0.604 ± 0.088	0.581 ± 0.118	0.187
Female	0.418 ± 0.091	0.409 ± 0.091	0.421 ± 0.092	0.725
Waist circumference (cm)				
Male	90.1 ± 8.6	89.8 ± 8.4	90.2 ± 8.77	0.766
Female	83.9 ± 10.4	82.4 ± 7.7	84.3 ± 11.1	0.613

*HTGP* hypertriglyceridemia-induced pancreatitis, *SAT* subcutaneous adipose tissue, *TAT* total adipose tissue, *VAT* visceral adipose tissue

**Table 3:** Uni-variate Logistic regression analysis of body composition parameters associated with the severity of hypertriglyceridemia-induced pancreatitis in male and female patients

Variables	Male		Female	
	*P* values	OR (95%CI)	*P* values	OR (95%CI)
Body mass index (> 26)	0.618	1.168 (0.634–2.154)	0.602	1.571 (0.287–8.595)
Muscle area (>median)	0.327	1.358 (0.736–2.504)	0.438	1.750 (0.426–7.190)
Muscle radiodensity (>median)	0.070	0.565 (0.304–1.049)	0.438	0.571 (0.139–2.348)
SAT area (>median)	0.327	1.358 (0.736–2.504)	0.147	3.020 (0.678–13.442)
SAT radiodensity (>median)	0.110	1.652 (0.892–3.060)	0.623	1.425 (0.347–5.851)
VAT area (>median)	0.377	0.759 (0.412–1.399)	0.147	3.020 (0.678–13.442)
VAT area/TAT area (>median)	0.377	0.759 (0.412–1.399)	0.438	1.750 (0.426–7.190)
Waist circumference (>median)	0.327	1.358 (0.736–2.504)	0.942	1.053 (0.262–4.224)

*HTGP* hypertriglyceridemia-induced pancreatitis, *SAT* subcutaneous adipose tissue, *TAT* total adipose tissue, *VAT* visceral adipose tissue

### Logistic regression analyses of risk factors for the severity of HTGP

Uni-variate Logistic regression analysis was applied to explore parameters associated with the severity of HTGP. Consequently, age, lipase, amylase, CRP, albumin, total cholesterol, HDL-C, Non-HDL-C, apoA-I and muscle radiodensity were associated with the severity of HTGP (Table 4). High Pearson correlations were found between non-HDL-C and total cholesterol (*r* = 1.000, *P* < 0.001), between lipase and amylase (*r* = 0.877, *P* < 0.001), and between apoA-I and HDL-C (*r* = 0.848, *P* < 0.001). Therefore, total cholesterol, amylase and HDL-C were removed from the following multi-variate Logistic analysis.

**Table 4:** Logistic regression analysis of parameters associated with the severity of hypertriglyceridemia-induced pancreatitis

Variables	Uni-variate analysis		Multi-variate analysis	
	*P* values	OR (95%CI)	*P* values	OR (95%CI)
Age	**0.027**	0.526 (0.298–0.930)	0.083	0.524 (0.253–1.087)
Sex	0.144	0.568 (0.266–1.214)		
Lipase	**0.017**	2.050 (1.134–3.704)	**0.033**	2.283 (1.070–4.873)
Amylase	**0.040**	1.808 (1.028–3.179)		
CRP (> 90 mg/L)	**< 0.001**	5.234 (2.767–9.899)	**0.005**	3.061 (1.407–6.659)
Albumin (< 35 g/L)	**< 0.001**	6.168 (3.033–12.546)	**0.004**	3.362 (1.492–8.823)
Triglyceride (≥22.4 mmol/L)	0.207	1.534 (0.790–2.979)		
Total cholesterol	**0.002**	2.479 (1.390–4.420)		
HDL-C	**< 0.001**	0.210 (0.111–0.397)		
LDL-C	0.124	1.553 (0.886–2.720)		
Non-HDL-C	**0.002**	2.512 (1.408–4.482)	**0.170**	1.724 (0.739–3.749)
ApoA-I (< 1.1 g/L)	**< 0.001**	6.495 (3.390–12.446)	**< 0.001**	5.126 (2.348–11.195)
ApoB	0.808	1.077 (0.592–1.959)		
Lipoprotein	0.885	1.043 (0.591–1.838)		

*ApoA-I* apolipoprotein A-I, *ApoB* apolipoprotein B, *CRP* C-reactive protein, *HDL-C* high-density lipoprotein cholesterol, *LDL-C* low-density lipoprotein cholesterol

Multi-variate Logistic analysis confirmed that low albumin (*P* = 0.004, OR = 3.362, 95%CI = 1.492–8.823) and apoA-I (*P* < 0.001, OR = 5.126, 95%CI = 2.348–11.195) as well as high CRP (*P* = 0.005, OR = 3.061, 95%CI = 1.407–6.659) and lipase (*P* = 0.033, OR = 2.283, 95%CI = 1.070–4.873) worsened the severity of HTGP (Table 3).

### Analyses of risk factors for occurrence of pancreatic necrosis

A total of 178 patients, comprising 37 female and 141 male patients, underwent enhanced CT scanning. Of them, 37 (6 women, 31 men) presented with pancreatic necrosis. By uni-variate Logistic regression analysis, the risk of pancreatic necrosis was shown to be associated with high levels of CRP and SAT radiodensity, and low levels of serum albumin, LDL-C, HDL-C, apoA-I and apoB (all *P* < 0.05, Table 5). By multi-variate Logistic analysis, low serum albumin (*P* = 0.003, OR = 4.154, 95%CI = 1.633–10.564), low LDL-C (*P* = 0.013, OR = 0.306, 95%CI = 0.120–0.778) and high SAT radiodensity (*P* = 0.007, OR = 3.592, 95%CI = 1.417–9.106) remained to be significant risk factors of pancreatic necrosis in patients with HTGP (Table 5).

**Table 5:** Logistic analysis of parameters associated with the risk of pancreatic necrosis in patients with hypertriglyceridemia-induced pancreatitis.

Variables	Uni-variate analysis		Multi-variate analysis	
	*P* values	OR (95%CI)	*P* values	OR (95%CI)
CRP (> 90 mg/L)	**0.014**	3.069 (1.254–7.513)	0.385	1.650 (0.534–5.103)
Albumin (< 35 g/L)	**< 0.001**	4.350 (1.952–9.691)	**0.003**	4.154 (1.633–10.564)
LDL-C	**0.004**	0.317 (0.145–0.692)	**0.013**	0.306 (0.120–0.778)
HDL-C	**0.004**	0.312 (0.140–0.693)	0.939	1.057 (0.257–4.349)
ApoA-I	**0.044**	0.460 (0.216–0.979)	0.734	0.846 (0.322–2.221)
ApoB	**< 0.050**	0.443 (0.197–0.999)	0.379	0.632 (0.227–1.759)
SAT radiodensity	**< 0.001**	3.864 (1.737–8.593)	**0.007**	3.592 (1.417–9.106)

*ApoA-I* apolipoprotein A-I, *ApoB* apolipoprotein B, *CRP* C-reactive protein, *HDL-C* high-density lipoprotein cholesterol, *LDL-C* low-density lipoprotein cholesterol, *SAT* subcutaneous adipose tissue

### Association of albumin, apo A-I, CRP and lipase with the length of hospital stay and clinical scoring parameters for patients with HTGP

Since low albumin and apo A-I and high CRP and lipase were shown to be risk factors for severity of HTGP, their associations with the length of hospital stay and clinical severity scoring parameters were further studied. Low albumin and apo A-I, as well as high CRP and lipase were all associated with a longer hospital stay and higher Ranson scores (all *P* < 0.05, Table 6). Specifically, HTGP patients with low serum albumin had a significantly longer hospital stay (median 18 days vs. 11 days, *P* < 0.001) and higher proportion of hospital stay exceeding 2 weeks (67.0% vs. 29.7%, OR = 4.801, 95% CI = 2.771–8.318, *P* < 0.001) as compared to those patients with a high serum albumin (Table 6). Notably, low serum albumin was associated with all the clinical severity scoring parameters, including higher APACHE II, BISAP, Ranson and Marshall scores (all *P* < 0.05, Table 6). In this study, there were 8 patients with BISAP scores ≥3, who all presented with low serum albumin concentration.

**Table 6:** Association of albumin, apoA-I, CRP, and lipase with the length of hospital stay and clinical scoring parameters for patients with hypertriglyceridemia-induced pancreatitis

Variables	Albumin (g/L)		ApoA-I (g/L)		CRP (mg/L)		Lipase (U/L)		Missing value
	< 35	≥35	< 1.1	≥1.1	< 90	≥90	low	high	
Length of hospital stay N (%)									
< 2 weeks	34 (33.0)	97 (70.3)	85 (48)	42 (73.7)	63 (70.0)	53 (40.8)	74 (64.9)	46 (40.0)	1
≥ 2 weeks	69 (67.0)	41 (29.7)	92 (52)	15 (26.3)	27 (30.0)	77 (59.2)	40 (35.1)	69 (60.0)	
*P* values	**< 0.001**		**< 0.001**		**< 0.001**		**< 0.001**		
OR (95%CI)	4.801 (2.771–8.318)		3.031 (1.568–5.859)		3.390 (1.916–5.998)		2.775 (1.624–4.742)		
APACHE II N (%)									
< 8	61 (64.2)	94 (83.2)	113 (70.6)	37 (86.0)	58 (80.6)	81 (68.1)	78 (82.1)	71 (66.4)	34
≥ 8	34 (35.8)	19 (16.8)	47 (29.4)	6 (14.0)	14 (19.4)	38 (31.9)	17 (17.9)	36 (33.6)	
*P* values	**0.002**		**0.041**		0.060		**0.011**		
OR (95%CI)	2.758 (1.443–5.268)		2.565 (1.015–6.483)		1.944 (0.966–3.911)		2.326 (1.202–4.503)		
BISAP N (%)									
< 3	85 (91.4)	107 (100)	150 (94.9)	39 (100)	69 (98.6)	106 (93.8)	88 (96.6)	101 (95.3)	42
≥ 3	8 (8.6)	0	8 (5.1)	0	1 (1.4)	7 (6.2)	3 (3.4)	5 (4.7)	
*P* values	**0.002**		0.360		0.125		0.637		
OR (95%CI)	0.443 (0.378–0.519)		0.794 (0.738–0.853)		4.577 (0.549–37.852)		1.419 (0.330–6.111)		
Ranson N (%)									
< 3	38 (40.9)	72 (67.3)	78 (49.7)	30 (75.0)	48 (67.6)	48 (42.9)	63 (71.6)	43 (40.2)	42
≥ 3	55 (59.1)	35 (32.7)	79 (50.3)	10 (25.0)	23 (32.4)	64 (57.1)	25 (28.4)	64 (59.8)	
*P* values	**< 0.001**		**0.004**		**0.001**		**< 0.001**		
OR (95%CI)	2.977 (1.670–5.307)		3.038 (1.391–6.635)		2.783 (1.494–5.184)		3.751 (2.052–6.857)		
Marshall N (%)									
< 2	74 (79.6)	104 (94.5)	136 (86.6)	37 (90.2)	66 (93.0)	96 (83.5)	81 (89.0)	91 (85.8)	39
≥ 2	19 (20.4)	6 (5.5)	21 (13.4)	4 (9.8)	5 (7.0)	19 (16.5)	10 (11.0)	15 (14.2)	
*P* values	**0.001**		0.543		0.061		0.506		
OR (95%CI)	4.450 (1.696–11.682)		1.428 (0.462–4.419)		2.613 (0.929–7.345)		1.335 (0.568–3.137)		

*APACHE* Acute Physiology and Chronic Health Evaluation, *ApoA-I* apolipoprotein A-I, *BISAP* Bedside Index for Severity in Acute Pancreatitis, *CRP* C-reactive protein, *HDL-C* high-density lipoprotein cholesterol

## Discussion

This study showed that low levels of albumin apoA-I and high levels of CRP and lipase upon admission were associated with the severity of HTGP and length of hospital stay, while none of the CT-based body composition parameters was linked to the severity of HTGP. Notably, low albumin concentration was also associated with pancreatic necrosis and all the severity scorings (APACHE II, BISAP, Ranson and Marshall) in patients with HTGP. To our knowledge, this is the first study suggesting that low albumin and apoA-I, which are routinely tested in the clinic, may serve as risk factors for moderately severe to severe HTGP.

Albumin has been suggested as a predictive factor for the severity of AP [27]. In a recent study including 708 patients with AP and an additional 477 patients from validation cohort, reported that albumin was an independent predictor for SAP and in-hospital mortality in AP patients [28]. Albumin has also been incorporated into some panels for predicting severity of AP [29, 30]. Serum albumin together with extrapancreatic fluid collections were suggested as the best indicator of severity of AP at the time of admission [31]. Combining blood urea nitrogen and albumin resulted in better prediction for SAP in pediatric patients [32]. Besides, low albumin is also helpful to predict organ failure in AP [33]. It has been shown that low serum albumin is independently associated with an increased risk of persistent organ failure and death in AP [34].

However, the association of albumin concentration with the severity in HTGP, has not been investigated before. The current study found that HTGP patients with albumin < 35 g/L had three times higher possibility to correlate with moderately severe or severe cases as compared to albumin ≥35 g/L. In HTGP, the deposition of free fatty acids hydrolyzed from TGs plays a crucial role in the mechanism of pancreatitis. Theoretically, a decrease of albumin, which can bind to the free fatty acids in the serum, may result in accumulation of free fatty acid, thus contributing to the inflammatory progression. Decrease of albumin may also be a consequence of the severe type of HTGP with higher amount of free fatty acid to be neutralized by albumin, and with increased vasopermeability allowing more albumin to permeate into the extravascular tissue space [35]. Besides, a decrease in albumin levels might also be relevant to the acute phase reaction due to the inflammatory response of HTGP.

Interestingly, this study showed that apoA-I alone achieved the highest AUC (0.786) to predict the severity of HTGP by ROC curves. With a balanced cutoff of 1.1 g/L, apoA-I predicted the severity of HTGP with a sensitivity and specificity of 0.725 and 0.784, respectively. Furthermore, apoA-I was revealed as an independent predictor for the severity of HTGP with an OR of 5.126. ApoA-I and apoB are the main carrier proteins of HDL and non-HDL, respectively. The predictive role of lipid and lipoprotein parameters in the severity of AP and its subtype, HTGP, has drawn attention recently, due to their accessibility in the clinic. For instance, the ratio of apoB to apoA-I, and HDL-C/LDL-C have also been postulated to correlate with the severity of AP [36, 37]. Other studies showed that apoA-I and HDL-C were negatively associated with the severity of AP [38, 39]. In HTGP, it has also been reported that apoB was predictive for the occurrence of the disease in patients with HTG [40]. Besides, a recent study has showed that higher total cholesterol was indicative of the more severe type of HTGP and less efficiency of plasmapheresis [41]. To our knowledge, this study was the first to indicate the predictive role of apoA-I for the severity of HTGP. However, the underlying mechanism remain to be unveiled.

Although TGs level was not associated with the severity of HTGP in this study, which is in line with the study from Yu et al. [42], therapy that decrease the level of TGs less than 5.6 mmol/L is considered key to improve the outcome of HTGP. Insulin, heparin, and plasmapheresis are possible treatments to reduce serum TGs levels during the acute episode. To prevent the re-occurrence of HTGP, it is also crucial to keep the level of TGs consistently < 5.6 mmol/L. Apart from the management of potential secondary factors that contribute to HTG, the treatment for HTG include weight loss, a very-low-fat diet, restricted carbohydrate and alcohol intake, fibrates, niacin and omega-3 fatty acids. Waylivra, an antisense oligo nucleotide inhibitor of apolipoprotein C- III, has recently been approved by the European Union for the treatment of FCS [6]. Besides, some new drugs targeting the TGs metabolism, including pemafibrate, AKCEA-APOCIII-LRX, AKCEA-ANGPTL3-LRX, ARO-APOC3 and ARO-ANGPTL3, have shown positive results in clinical trials [6, 9].

CRP and lipase levels upon admission were predictive for the severity of HTGP in this study. The result of the predictive role of CRP was consistent with the report from Yu and colleagues, in which 159 HTGP Chinese patients were retrospectively studied and high CRP and BMI were postulated as risk factors for severe HTGP [42]. CRP is one of the most widely utilized biomarkers in clinical practice for AP. CRP levels of > 150 mg/L 48 h after onset of symptoms have a high sensitivity for predicting severity of AP [43]. A rise of > 90 mg/dL from admission or an absolute value of > 190 mg/dL at 48 h predicted severe disease [44]. However, it is generally considered that the initial CRP value (at time of admission) is too early to be predictive for the severity of AP [45]. Lipase has been reported to be significantly higher in severe HTGP than in the non-severe group, although it failed to represent an independent factor for severity of HTGP [42].

The body composition parameters, especially VAT, have been reported to correlate with the severity of AP. Due to the involvement of lipoprotein metabolism in HTGP, this study also investigated whether body composition parameters were associated with the severity of HTGP. Intriguingly, no association was found between the severity of HTGP and any of these body composition parameters, including the CT-measured muscle area, muscle radiodensity, SAT area/radiodensity, VAT area, and body mass index and waist circumference. In comparison, VAT area was reported to correlate with the severity of HTGP [23]. Several factors may contribute to the discrepancy. Firstly, the present study noticed that gender has a significant impact on all body composition parameters evaluated, in line with previous reports [46]. Therefore, the cut-off level of each body composition parameter and the subsequent statistical analysis were gender dependent in the present study. Secondly, the present study included HTGP with any severity, while the previous study only included moderately severe to severe patients from the intensive care unit [23]. Lastly, there is a difference of sample size between the two studies.

Pancreatic necrosis is a parameter for CT-based evaluation of the severity of AP, and is closely associated with morbidity and mortality in AP [47]. The management of pancreatic necrosis has been highlighted due to its significance for the outcome of AP [48]. The association between body composition and mortality in AP with pancreatic necrosis has been investigated, concluding that loss of skeletal muscle density within the first month after initial admission was significantly associated with increased mortality [49]. It has been shown that patients with HTGP had a higher incidence of pancreatic necrosis as compared to AP with other etiologies [50]. In HTGP, excess TGs are hydrolyzed by lipase from pancreatic acinar cells to produce FFAs, which in turn cause acinar necrosis [3]. This study suggested that low serum albumin and LDL-C and high SAT radiodensity were associated with pancreatic necrosis in patients with HTGP.

### Study strengths and limitations

This study, which enrolled 242 patients with HTGP, for the first time revealed that albumin and apoA-I, may serve as novel biomarkers for the severity of HTGP. There are several limitations of the study. Firstly, this study was carried out retrospectively. Although there was an auto-dilution procedure of blood samples before chemical analysis with a routine method in the clinic, HTG may have impact on the determination of other laboratory parameters. Secondly, this study was not able to investigate the causes of HTG in patients, including the proportion of FCS.

## Conclusions

This study showed that low levels of albumin and apoA-I, as well as high levels of CRP and lipase upon admission, were associated with the severity of HTGP and the length of hospital stay. Albumin and apoA-I may serve as novel biomarkers for the severity of HTGP. Besides, HTGP patients with low albumin should be kept under close surveillance during the disease progression. Prospective validation of the predictive role of albumin and apoA-I for the severity of HTGP are needed before translating the finding to clinical practice.

## Data Availability

All relevant data and materials are included in the manuscript. For the full detailed data, please contact the corresponding author.

## Abbreviations

AP: Acute pancreatitis
APACHE: Acute Physiology and Chronic Health Evaluation
ApoA-I: Apolipoprotein A-I
ApoB: Apolipoprotein B
AUC: Areas under the curves
BISAP: Bedside Index for Severity in Acute Pancreatitis
BMI: Body mass index
CI: Confidence interval
CRP: C-reactive protein
CT: Computed tomography
FCS: Familial chylomicronemia syndrome
HDL-C: High-density lipoprotein cholesterol
HTG: Hypertriglyceridemia
HTGP: Hypertriglyceridemia-induced pancreatitis
HU: Hounsfield units
LDL-C: Low-density lipoprotein cholesterol
MCTSI: Modified CT severity index
OR: Odds ratio
ROC: Receiver operating characteristic
SAT: Subcutaneous adipose tissue
TAT: Total adipose tissue
TG: Triglyceride
VAT: Visceral adipose tissue
